# Macular thickness measurements in healthy Norwegian volunteers: an optical coherence tomography study

**DOI:** 10.1186/1471-2415-10-13

**Published:** 2010-05-13

**Authors:** Alexandra Wexler, Trond Sand, Tor B Elsås

**Affiliations:** 1 Department of Ophthalmology, St. Olavs University Hospital, Trondheim, Norway; 2 Department of Neuroscience, Norwegian University of Science and Technology, Trondheim, Norway; 3 Department of Neurology and Clinical Neurophysiology, St. Olavs University Hospital, Trondheim, Norway

## Abstract

**Background:**

Ethnic, intersubject, interoperator and intermachine differences in measured macular thickness seem to exist. Our purpose was to collect normative macular thickness data in Norwegians and to evaluate the association between macular thickness and age, gender, parity, and contraception status.

**Methods:**

Retinal thickness was measured by Stratus Optical Coherence Tomography in healthy subjects. Mean macular thickness (MMT) was analyzed by repeated measures ANOVA with three dependent regional MMT-variables for interaction with age, gender, parity and oral contraception use. Exploratory correlation with age by the Pearson correlation test, both before and after stratification by gender was performed. Differences in MMT between older and younger subjects, between oral contraception users and non-users, as well as parous and nulliparous women were studied by post-hoc Student's t-tests.

**Results:**

Central MMT in Norwegians was similar to values earlier reported in whites. MMT in central areas of 1 and 2.25 mm in diameter were higher in males than in females. In younger subjects (≤43 years) differences in MMT between genders were larger than in the mixed age group, whereas in older subjects (>43 years) the small differences did not reach the set significance level. No differences were found in minimal foveolar thickness (MMFT) between the genders in any age group.

Mean foveal thickness (1 mm in diameter) was positively associated with age in females (r = 0.28, p = 0.03). MMFT was positively associated with age in all groups and reached significance both in females and in mixed gender group (r = 0.20, p = 0.041 and r = 0.26, p = 0.044 respectively).

Mean foveal thickness and MMFT were significantly higher in parous than in nulliparous women, and age-adjusted ANOVA for MMFT revealed a borderline effect of parity.

**Conclusions:**

Age and gender should be taken into consideration when establishing normal ranges for MMT in younger subjects. The gender difference in retinal thickness in young, but not older adults suggests a gonadal hormonal influence. The possible association between parity and retinal structure and its clinical relevance, should be studied further.

## Background

In vivo qualitative and quantitative imaging of the retina by the optical coherence tomography (OCT) [1] is non-invasive, obtainable and reproducible even on non-dilated eyes [2-4] in both healthy subjects and in patients with macular pathology [5,6].

Macular aging involves alterations in its function, structure [7] and blood supply [8], which are partly induced by chronic low-grade inflammation [9]. Complex multifactorial genetic and environmental factors may accelerate the aging process or trigger a progressive and irreversible loss of central vision [10], as in age-related macular degeneration [11,12]. Some of these factors seem to be modulated by gonadal sex hormones [13-15].

Sex-related differences exist in both healthy and diseased eyes [16,17], and several sight threatening retinal conditions like age-related macular degeneration and idiopathic macular holes have been associated with the female gender and reproductive history [18-20]. It has been suggested that the macula in females, being thinner, is more vulnerable than in males.

However, there is inconsistency as to whether mean macular thickness (MMT) varies with age and gender in published papers. Both gender specific sex hormones and age related hormonal changes in women are known to influence macular function [21,22]. Although there is a growing body of evidence that estrogens influence maintenance of retinal function and integrity [23,24], little is known about their effects on MMT measured by the OCT.

Ethnic differences exist in the prevalence of age-related macular degeneration [25], gonadal hormone levels in women [26] and in MMT [27-30], and may explain some of the variations in the published literature. Since the prevalence of early age-related maculopathy appears to be higher in the urban Norwegian population than in other populations [31] and that menopause possibly occur earlier in Norwegians (about 50 years) than in Europe (about 54 years) [32-35], we hypothesized that MMT measurements in Norwegians could differ from measurements in other populations. There are also intersubject, interoperator and intermachine variability in measured MMT, even when identical OCT versions are being used [36].

The purpose of the present study was to collect normative data on the Stratus OCT in Norwegians. We also assessed the effect of age, gender, parity and the use of oral contraception on macular thickness in our study sample.

## Methods

Subjects were prospectively recruited from students and staff at St. Olavs University Hospital. Inclusion criteria were best corrected visual acuity (Humphery automatic refractor HARK 597, Dublin, CA) better than 0.8 with spheric equivalent of ±6, no current medical eye history (uncomplicated refractive surgery >2 years prior to enrollment was accepted), no evidence of pathology on slit lamp microscopy with 90-diopter lens, no significant lens opacities and normal intraocular pressure. Subjects with diabetes or systemic inflammatory conditions were not included.

Initially 258 phakic, healthy-appearing eyes of 129 subjects were included. Fifteen single eyes of 15 subjects were excluded because they applied diclofenac or dexamethasone topically for three days in one eye (parallel study). Eighteen subjects (bilateral scans) and 18 single eyes (of 16 subjects) were excluded due to low OCT signal, badly defined interfaces, alignment problems and/or missing data on the scan. Two subjects were excluded in spite of good OCT quality; one subject had eye symptoms with "foggy sight" despite normal eye examination, another had had heavy head trauma several years prior to examination.

Overall 185 OCT scans of 107 subjects, 78 bilateral and 29 unilateral were eligible for inclusion. Only one eye of each subject was included, laterality was randomly chosen (where bilateral scans were available).

All subjects gave their informed consent. The study was approved by the Regional Ethics Committee in May 2005. It was conducted in accordance with the Declaration of Helsinki recommendations. Subjects were included between September 2007 and September 2008.

Both eyes were examined and bilateral OCT (Optic Coherence Tomography STRATUS, Carl Zeiss Meditec, Inc., Dublin, CA) scans were obtained by a single operator on all eyes. Medical history was taken during the session, which included an optional interview about the use of hormonal contraception (44 of 62 women participated) and childbirth (57 of 62 women participated). If subjects consented to an extended examination, a multifocal electroretinography was also performed for future publications.

OCT was recorded in Macular Thickness Protocol (software v.5.0.1). Spherical values closest to subject's refraction were adjusted in the OCT, even though studies had shown that this did not affect macular thickness measurements significantly [2,37]. A few myopic soft contact lens users were allowed to keep the correction on. The room was darkened to gain maximal undilated pupil size and volunteers were asked to gaze at the internal fixation mark within the OCT, while six radial retinal scans were taken.

The scans were obtained at equally spaced angular orientation centered on the foveola. MMT is generated by the OCT software algorithms by calculating the distance between the vitreoretinal interface and the boundary between the inner and outer photoreceptor segment in microns. Each 6 millimeter long scan takes measurements at 512 points, with higher density near the foveola. Mean macular thickness (MMT) is then calculated automatically and reported.

Pharmacological dilatation was not routinely applied, as it is not a necessary procedure for Stratus OCT [3,4]. However, noisy scans were retaken after dilatation with tropicamide 0.5% one drop in each eye if subjects consented (after removing contact lenses). Eye movements, fixation losses and blinks prolonged the procedure, while undilated pupils [38] and soft contact lenses [39] deteriorated the signal strength. Barkana et al. [40] found that signal strength did not affect any measured MMT parameters when scans with signal strength ≥4 (out of maximum ten) were included (using the fast macular protocol) [40]. Moreover, Muscat et al. [41] showed that even considerably degraded signal strength produced accurate, precise and reproducible MMT measurements.

Scans with well defined interfaces and signal strength better than three were included. Measured MMT could vary with axial length and refraction by altering the area measured in a scan, because the default axial length in the STRATUS OCT is set to 24.45 mm. A longer axial length and myopia results in axial magnification and a larger circle area would be measured [37]. However, since peripheral MMT measurements are more susceptible to axial magnification than central [37], and there is high concordance in measured MMT in the central areas by the shorter (3.45 mm) and the longer (6 mm) scan length mapping protocols [42], the shorter (3.45 mm) scan length was chosen (even though no high myopes were included).

**Figure 1:**
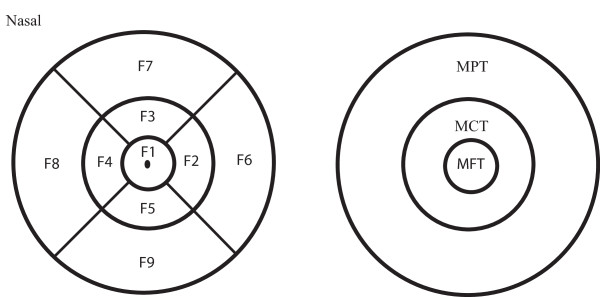
**Macular areas on the Stratus OCT scan**. Outer diameter of the outer, middle and inner rings are 3.45, 2.22 and 1 millimeters respectively. The foveal point in the center of F1 represents the mean minimal foveolar thickness (MMFT = F0). Mean foveal thickness (MFT) is measured in area F1. Regional thickness variables for regions MCT (mean central thickness) = (F2 + F3 + F4 + F5)/4, MPT (mean peripheral thickness) = (F6 + F7 + F8 + F9)/4 and MTT (mean total thickness) = (F1 + F2 + F3 + F4 + F5 + F6 + F7 + F8 + F9)/9 were calculated.

In this protocol the macula is divided into 9 areas: Mean foveal thickness (MFT = F1) from a central macular area of one millimeter in diameter, the inner ring (F2-F5) and the outer ring (F6-F9), each divided into four quadrants. The outer ring diameters measured 2.22 and 3.45 millimeters respectively. Regional variables MCT (mean central thickness) and MPT (mean peripheral thickness) were defined by averaging middle (MCT = (F2 + F3 + F4 + F5)/4) and outer (MPT = (F6 + F7 + F8 + F9)/4) ring data (Fig. 1). Mean total averaged macular thickness (MTT) was calculated by averaging the nine macular areas (F1 + F2 + F3 + F4 + F5 + F6 + F7 + F8 + F9)/9. The inner region (MFT = F1) and mean minimal foveolar thickness (MMFT = F0) were also analyzed.

Participants were categorized in 11 partly overlapping subgroups according to age, parity, gender and oral contraception use. Parity was defined by delivering a live-borne child, contraception users were defined by use of oral contraception for at least three month prior to inclusion. No pregnant women were included. These subgroups are described, together with their mean OCT signal strength in Table 1.

**Table 1:** Subjects in partly overlapping subgroups according to age, parity, gender and contraception status.

Group	Number of subjects	Mean age (SD) in years	Age range	Mean OCT signal strength(SD)
All participants	107	42.4(11.8)	21-63	6.3(1.7)
Males	45	39.2(12.0)	22-63	6.0(1.9)
Females	62	44.7(11.2)	21-61	6.5(1.6)
Younger* males	29	31.6(6.3)	22-42	6.3(2.0)
Younger females	27	33.8(7.0)	21-43	6.1(1.5)
Older** males	16	52.9(5.9)	44-63	5.4(1.5)
Older females	35	53.0(4.7)	44-61	6.7(1.6)
Parous women	34	46.7(8.4)	28-60	6.7(1.4)
Nulliparous womean	23	40.0(14.3)	21-61	6.2(1.7)
Women using oral contraception	10	33.5(9.5)	21-53	6.0(1.8)
Women not using oral contraception	33	48.0(10.3)	24-60	6.7(1.4)

OCT: optical coherence tomography. *age ≤43 (mean age for all participants), **age >43

One OCT scan of each subject was included, laterality was randomly chosen if bilateral scans were available, so that equal amount of right (n = 54) and left (n = 53) eyes were included. Signal strength was 4 or 5 in 38 scans, 6 or 7 in 43 scans and 8 to 10 in 26 included scans. MMT in the above described macular areas were analyzed and compared between the groups.

Regional variables MFT, MCT and MPT were analyzed with repeated measures ANOVA with age as covariate and gender as the grouping factor. Within-subject factors were assessed with multivariate repeated measures analysis. In women we performed two additional ANOVAs, one with parity and another with contraception use as grouping factors.

The associations between MMFT (foveola), age and either gender, parity, or contraceptives were studied by three separate ANOVAs with MMFT as the dependent variable.

MMT was also explored for statistical association with age in three separate groups (all 107 subjects, male subgroup and female subgroup) with the Pearson correlation test for the five macular variables (MMFT, MFT, MCT, MPT and MTT).

MMT in these five macular regions was analyzed with independent sample Student's t-tests for differences between the genders in three different groups: comparing males with females, younger males with younger females and older males with older females. MMT in parous women was compared with nulliparous women, and oral contraception users were compared with non-users by the same tests.

Data were analyzed in SPSS 16. All tests were two-sided and a p-value < 0.05 was considered statistically significant.

## Results

Normal mean regional MMT-values are reported in Table 2. Males had higher MMT values than females in central macular regions except MMFT (Table 2). In repeated measures ANOVA gender did affect MMT significantly (p = 0.001; Table 3). Significant interaction between region and gender was found (p = 0.009; Table 3).

**Table 2:** MMT (SD) (in micron) in five regions related to gender in 107 healthy subjects.

Macular regions	Both genders (n = 107)	Males (n = 45)	Females (n = 62)	**p-value**^**1**^
F0 (MMFT)	178(22)	179(21)	177(20)	0.47
Inner ring F1(MFT)	213(16)	218(16)	209(15)	0.002
Middle ring (MCT)	272(15)	279(15)	268(13)	<0.0005
Outer ring (MPT)	274(14)	277(16)	272(13)	0.14
Total region (MTT)	267(13)	271(14)	263(12)	0.003

MMT: mean macular thickness MMFT: mean minimal foveolar thickness, MFT: mean foveal thickness, MCT: mean central thickness, MPT: mean peripheral thickness, MTT: mean total thickness. The outer diameter of the inner, middle and outer rings is 1, 2.22 and 3.45 millimeters respectively. For further definition of macular regions see Fig.1. ^1^Student's t-test (males vs. females)

**Table 3:** Repeated measures ANOVA with three dependent regional MMT-variables (MFT, MCT and MPT).

	F (df)	p
**Between subjects**		
Age^1^	0.28(1)	0.60
Gender^1^	11.2(1)	0.001
Age^2 ^(females)	2.8(1)	0.10
Parity^2^	1.8(1)	0.19
Contraception^3^	0.58(1)	0.45

**Within subjects**^**4**^		
Region^1^	153 (2)	<0.0005
Region*age^1^	3.5 (2)	0.03
Region*gender^1^	4.8 (2)	0.009
Region*parity^2^	1.0 (2)	0.38
Region*contraception^3^	1.8 (2)	0.17

^1^First ANOVA (age and gender), ^2^Second ANOVA (age and parity (yes/no), females), ^3^Third ANOVA (age and oral contraception (yes/no), females). ^4^Multivariate repeated measures test. MMT (mean macular thickness) in three regions; MFT: mean foveal thickness (inner ring), MCT: mean central thickness (middle ring), MPT: mean peripheral thickness (outer ring). The outer diameter of the inner, middle and outer rings is 1, 2.22 and 3.45 millimeters respectively. For further definition of macular regions see Fig.1

**Figure 2:**
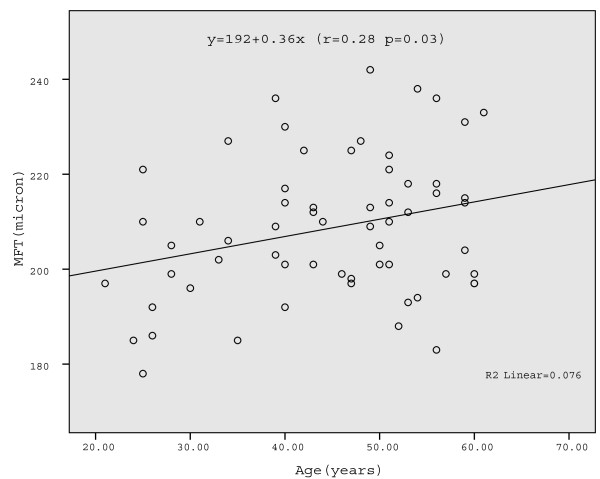
**Mean foveal macular thickness (MFT) related to age in females**. Linear regression is shown (Pearson correlation r = 0.28, p = 0.03).

In repeated measures ANOVA age was not significantly associated with MMT, although we observed a trend when females were analyzed separately (p = 0.1); Table 3). In the exploratory correlation analysis of MMT, we found a small but significant positive correlation between MFT and age in females (r = 0.28 p = 0.03) (Table 4; Fig. 2), and between MMFT and age in both females (r = 0.26 p = 0.044) and mixed-gender group (r = 0.20 p = 0.041) (Table 4). However, r-values were generally small, explaining less than 8% of the variance.

**Table 4:** Pearson correlation coefficients between age and mean macular thicknesses in five regions.

	Both genders (n = 107)	Males only (n = 45)	Females only (n = 62)
F0 (MMFT)	0.20^**a**^	0.18	0.26^**a**^
Inner ring F1 (MFT)	0.08	0.03	0.28^**a**^
Middle ring (MCT)	-0.08	-0.22	0.21
Outer ring (MPT)	-0.07	-0.18	0.01
Total region (MTT)	-0.06	-0.19	0.18

MMFT: mean minimal foveolar thickness, MFT: mean foveal thickness, MCT: mean central thickness, MPT: mean peripheral thickness, MTT: mean total thickness. The outer diameter of the inner, middle and outer rings is 1, 2.22 and 3.45 millimeters respectively. For further definition of macular regions see Fig.1. ^a^p < 0.05

**Figure 3:**
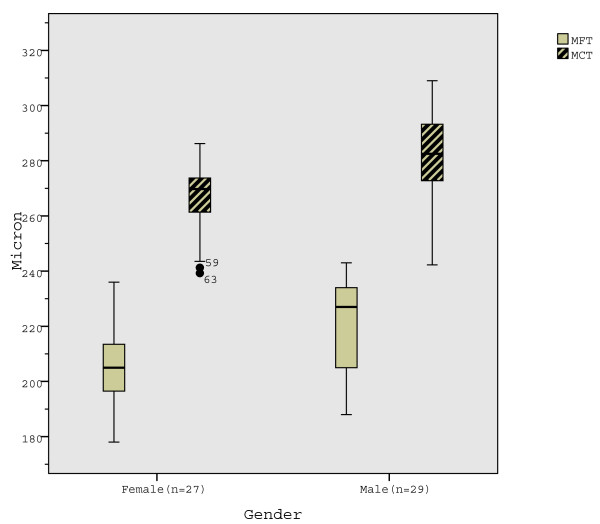
**Mean foveal thickness (MFT) and mean central thickness (MCT) in younger (≤43 years) males and females**. MFT and MCT are significantly thicker in males in younger subjects.

Differences between the genders in all central MMT regions (except MMFT) were significant in younger subjects (Table 5; Fig. 3) and in mixed-age group (Table 2), measuring higher values in males. Differences in MMT were non-significant in older subjects (Table 5).

**Table 5:** MMT (in micron(SD)) by gender, related to age (younger/older than 43) in macular regions.

	Males	Females	**p-value**^**1**^
Younger group (age ≤ 43)	(n = 29)	(n = 27)	
F0 (MMFT)	180(23)	173(19)	0.27
Inner ring F1 (MFT)	219(18)	206(15)	0.002
Middle ring MCT	281(16)	266(13)	<.0005
Outer ring MPT	279(17)	272(14)	0.092
Total region MTT	274(15)	262(12)	0.003
Older group (age > 43)	(n = 16)	(n = 35)	
F0 (MMFT)	179(18)	179(20)	0.98
Inner ring F1 (MFT)	211(15)	211(15)	0.22
Middle ring MCT	274(14)	269(12)	0.25
Outer ring MPT	272(14)	273(13)	0.84
Total region MTT	267(13)	264(12)	0.54

MMT: mean macular thickness, MMFT: mean minimal foveolar thickness, MFT: mean foveal thickness, MCT: mean central thickness, MPT: mean peripheral thickness, MTT: mean total thickness. The outer diameter of the inner, middle and outer rings is 1, 2.22 and 3.45 millimeters respectively. For further definition of macular regions see Fig.1. ^1^Student's t-test (males vs. females).

**Figure 4:**
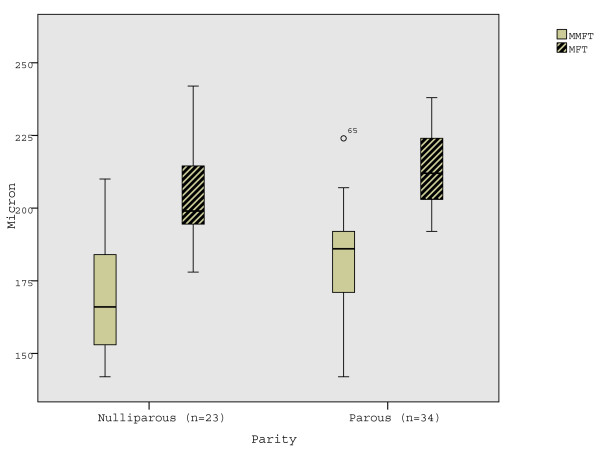
**Mean minimal foveolar thickness (MMFT) and mean foveal thickness (MFT) in parous and nulliparous women**. MMFT and MFT are significantly thicker in parous women.

Age-adjusted ANOVA for MMFT revealed a borderline effect for parity (F = 3.5, p = 0.066). No significant interaction between region and parity was observed (Table 3). However, with post-hoc Studen's t-tests MMFT, and MFT were observed to be significantly higher in parous compared to nulliparous women (Table 6; Fig. 4). Differences in MMT between women with and without oral contraception were not found.

**Table 6:** MMT (SD) (in micron) related to parity among women in macular regions.

	Parous women (n = 34)	Nulliparous women (n = 23)	**p-value**^**1**^
F0 (MMFT)	182(16)	170(21)	0.016
Inner ring F1 (MFT)	213(12)	205(17)	0.049
Middle ring MCT	270(12)	267(15)	0.36
Outer ring MPT	275(12)	269(14)	0.08
Total region MTT	266(11)	261(13)	0.12

MMT: mean macular thickness, MMFT: mean minimal foveolar thickness, MFT: mean foveal thickness, MCT: mean central thickness, MPT: mean peripheral thickness, MTT: mean total thickness. The outer diameter of the inner, middle and outer rings is 1, 2.22 and 3.45 millimeters respectively. For further definition of macular regions see Figure 1. ^1^Student's t-test (parous vs. nulliparous women).

## Discussion

Our measurements of MMT in central macular areas are in good agreement with other studies conducted on the Stratus OCT in whites. MMFT (foveola) was 177(SD 20) and MFT (fovea) 209 (15) in the female group while Liew et al. [43] found thicknesses of 178(23) and 212(19) in same areas in females aged 17 - 50 years. Chamberlain et al. [44] reported MFT of 210.3 (21) in Australian whites aged 50-80 years and Chan et al. [45] reported foveal MMT of 212(20) in mixed gender group, which is similar to our measured MFT of 213(16) in the mixed gender group. Evaluation of exact MMT in the more peripheral areas is difficult unless the same scan length is being used. MMT measurements in Norwegians do not seem to differ from earlier reported data on the Stratus OCT in whites.

Normal aging seems to affect macular cone function [46] but not foveal cone density [47]. Most [44,45,48-54] but not all [28,55] studies on the OCT did not report an association between foveal thickness and age in mixed gender group, which is in agreement with our results for MMT. However, our data in Table 4 suggest an increase in foveolar thickness (MMFT) with increasing age, which also confirms Kashani et al.'s [30] observation.

How can an age-related increase in MMT be explained? Foveolar cones rely solely on choriocapillar circulation to satisfy their high metabolic demands, lacking retinal capillaries [11]. Macular retinal [8] and foveolar choroidal microcirculation decline with age [56], which may impair the susceptible fovea preferentially faster [11,57]. Also aging-related para-inflammatory responses [9] may increase interstitial volume by impairing the blood-retina barrier [9,58], possibly resulting in higher MMT measured by the OCT.

Bjornsson [31] reported higher prevalence of early age- related maculopathy (EAMD) in Norwegians compared with other ethnic populations, which might suggest the age-related MFT increase we observed could be attributed to genetic or local environmental factors [30,43,51,59,60]. However, this is less likely because our MMT values were similar to those reported in other white ethnic groups. Moreover, we found that MFT did not increase with age in males as it did in females, suggesting that hormonal factors might explain the association between age and MMT.

Most OCT studies describe gender differences in MFT [28,45,48,49,53,54] or in several macular areas [52,55]. Only a few studies did not find gender differences in MMT [50,61,62]. We found gender differences in MMT in almost all macular areas in younger but not in older subjects, which suggest that the sexual dimorphism of the human retina may depend on gonadal hormone levels. Indeed, estrogen receptors seem to protect the retina against age-inflicted injury [23,24], partly by inhibiting lipid peroxidation [15,63,64]. Exposure to endogenous oestrogens has been associated with lower risk for EAMD [18,19], and exogenous estrogens seem to protect against late age-related degeneration [14]. It may accordingly be speculated that subclinical age-related para-inflammation may accelerate with declining estrogen levels and possibly explain the age-related MFT increase on the OCT in women.

Parity seems to lower estrogen levels in parous premenopausal compared with nulliparous women [65], partly by altering the sensitivity to sex hormones [66,67]. Reproductive history factors like long and/or irregular menstrual cycles, early menopause, pregnancy losses and multiparity are associated with higher risk for cardiovascular disease [68]. Hence, the trend towards an association between MMT and parity (Table 6) can possibly be related to a combination of hormonal and cardiovascular risk factors.

Why did we not find any effect of contraceptives on MMT? In the present study contraception use was defined by as a regular oral intake for at least three months prior to inclusion. Neither type of oral contraceptives nor their cumulative time of use were registered. We did not ask about the interdependendt factors as hormonal replacement therapy use, menopause, number of years from last pregnancy, lost pregnancies and multiparity. It is accordingly possible that a biologially relevant effect of oral contraceptives on MMT could have been demonstrated in a larger study with a suitable for this purpose design. Anyway, it should be noted that rather short-lasting current use of contraceptives did not seem to affect retinal thickness.

To make participation in our study convenient, a few younger myopic subjects kept their contact lenses on during the OCT measurement. This could have affected the measured MMT. Youm et al. [39] found a small increase (1.4 μ (SD 0.5)) in measured retinal nerve fiber layer (RNFL) (using Fast Protocol) when contacts were removed. However, this small effect is less than 2% of the MMT. In addition, RNFL measurements, unlike MMT measurements, are very susceptible to low scan quality (signal- to noise ratio and signal strength) [69,70]. Hence, we believe that the potential effect of contact lens use during our measurement of MMT is very small if at all present. However, we can not exclude the possibility of a small contact lens effect, and also this topic should be addressed in a future study.

## Conclusions

OCT measurements in healthy Norwegians did not differ from measurements in other whites. Differences in MMT between the genders in younger but not in older subjects were observed. Minimal foveolar thickness in both genders and female's foveal thickness were positively associated with increasing age, while a trend towards an effect of parity on central MMT was revealed. Age and gender should be taken into consideration when establishing normal ranges for MMT in younger subjects. Further studies are needed in women to increase our understanding of the possible association between MMT, parity, aging and hormonal status.

## Competing interests

The authors declare that they have no competing interests.

## Authors' contributions

TBE suggested the initial concept and together with AW was responsible for planning the study design. AW carried out the data collection including OCT measurements. TS helped with the statistical analysis and interpretation. All authors have participated in the writing and approval of the final manuscript.

## Pre-publication history

The pre-publication history for this paper can be accessed here:

http://www.biomedcentral.com/1471-2415/10/13/prepub
